# GWAS Discovery of Candidate Genes for Yield-Related Traits in Peanut and Support from Earlier QTL Mapping Studies

**DOI:** 10.3390/genes10100803

**Published:** 2019-10-12

**Authors:** Juan Wang, Caixia Yan, Yuan Li, Chunjuan Li, Xiaobo Zhao, Cuiling Yuan, Quanxi Sun, Shihua Shan

**Affiliations:** 1 Genetic Breeding Group, Shandong Peanut Research Institute, Qingdao 266000, China; wangjuan_1984@163.com (J.W.); cxyan335@sina.com (C.Y.); peanutlab@163.com (C.L.); zhaoxiaoboqd@126.com (X.Z.); yuancl1982@163.com (C.Y.); squanxi@163.com (Q.S.); 2 Computational Biology and Biological Physics, Astronomy and Theoretical Physics, Lund University, 24012 Lund, Sweden; Yuan.Li@thep.lu.se

**Keywords:** peanut, GBS-GWAS, QTLs, co-localization

## Abstract

Peanut (*Arachis hypogaea* L.) is one of the most important oil crops worldwide, and its yet increasing market demand may be met by genetic improvement of yield related traits, which may be facilitated by a good understanding of the underlying genetic base of these traits. Here, we have carried out a genome-wide association study (GWAS) with the aim to identify genomic regions and the candidate genes within these regions that may be involved in determining the phenotypic variation at seven yield-related traits in peanut. For the GWAS analyses, 195 peanut accessions were phenotyped and/or genotyped; the latter was done using a genotyping-by-sequencing approach, which produced a total of 13,435 high-quality single nucleotide polymorphisms (SNPs). Analyses of these SNPs show that the analyzed peanut accessions can be approximately grouped into two big groups that, to some extent, agree with the botanical classification of peanut at the subspecies level. By taking this genetic structure as well as the relationships between the analyzed accessions into consideration, our GWAS analyses have identified 93 non-overlapping peak SNPs that are significantly associated with four of the studied traits. Gene annotation of the genome regions surrounding these peak SNPs have found a total of 311 unique candidate genes. Among the 93 yield-related-trait-associated SNP peaks, 12 are found to be co-localized with the quantitative trait loci (QTLs) that were identified by earlier related QTL mapping studies, and these 12 SNP peaks are only related to three traits and are almost all located on chromosomes Arahy.05 and Arahy.16. Gene annotation of these 12 co-localized SNP peaks have found 36 candidates genes, and a close examination of these candidate genes found one very interesting gene (*arahy.RI9HIF*), the rice homolog of which produces a protein that has been shown to improve rice yield when over-expressed. Further tests of the *arahy.RI9HIF* gene, as well as other candidate genes especially those within the more confident co-localized genomic regions, may hold the potential for significantly improving peanut yield.

## 1. Introduction

As one important source of edible oil, the cultivated peanut (*Arachis hypogaea* L.) has been planted in more than 100 countries (FAOSTAT; http://faostat.fao.org) [1], including China, which has become the largest producer and exporter of peanut in the world [2]. Nevertheless, there is still a huge market demand for peanut, which may be resolved with genetic improvement of yield-related traits [3,4]. 

Yield-related traits, such as hundred-seed/pod weight and mature pod number per plant, are mostly quantitative traits, which have been found to be governed by multiple loci and are influenced by environmental factors [5,6]. Understanding the genetic base of yield-related traits is the most important prerequisite for peanut genetic improvement and such understanding has been greatly advanced, especially through approaches that search for quantitative trait loci (QTL). Over the past two decades, QTLs for yield-related traits have been identified mostly by traditional bi-parental QTL mapping [6,7,8,9] and relatively recently by genome-wide association studies (GWAS) [10]. GWAS analysis has some significant advantages over traditional QTL mapping; for example, the much greater historical recombination and genetic diversity that are usually embedded within the studied plant lines for the former significantly increase the mapping resolution of this time-efficient method and make it rather easy for minor effect genes to be detected [11].

GWAS has been used more and more often to study the genetic base of important traits in peanut [12,13,14,15], but most of these studies are restricted by the limited number of markers that can be used. In the past decade, the fast developing next-generation sequencing (NGS)-related technologies, such as reduced-representation sequencing including genotyping-by-sequencing (GBS), restriction-site-associated DNA sequencing (RAD-seq), specific-locus amplified fragment sequencing (SLAF-seq), and single nucleotide polymorphisms (SNP) array, have generated a large amount of SNPs that provide us a great opportunity to use GWAS for studying the genetic base of crop traits [8,10,15,16]. Now, NGS-based GWAS has been proved to be a cost-effective tool with great resolution for detecting important QTLs, for example, in maize [17], soya bean [18,19], upland cotton [20,21], and common bean [22]. In peanut, a high-density SNP array ‘Axiom_*Arachis*’ with 58K SNPs that has a genome-wide coverage has been developed from 41 peanut accessions and some wild peanut relatives [15]; this SNP array has the potential to be used for peanut genotyping, which can further help carry out GWAS analyses for dissecting important agronomic traits in peanut. Zhang et al. [10] identified 17,338 high-quality SNPs using the SLAF-seq method, and based on these high-quality SNPs, they have also implemented GWAS analyses to dissect the molecular basis of domestication-related agronomic traits within 158 peanut accessions.

To further explore the genetic resources embedded within cultivated peanut, in the present study, 195 peanut accessions were genotyped using the GBS method, which has been made possible by the recent release of peanut whole genome sequence data (https://www.peanutbase.org/) [23,24]. These genotype data together with the phenotype data that are collected from the analyzed accessions were used to perform GWAS analyses for identifying genomic regions that are significantly associated with seven peanut yield-related traits. The identified associated regions were then compared to the QTL loci reported in earlier QTL mapping/GWAS studies (using different sets of peanut accessions comparing to the present study) [6,7,9,12,13,14], and co-localized regions received close examination.

## 2. Materials and Methods 

### 2.1. Plant Materials

In this study, a total of 195 peanut accessions were collected from 20 provinces that represent the peanut cultivation areas in China (Figure S1), among which, 82 belong to *A. hypogaea* var. *hypogaea*, 30 var. *hirsuta*, 56 var. *vulgaris*, 18 var. *fastigiata* plus nine irregular types (Figure S1 and Table S1). The irregular types were not members of any previous defined peanut botanical varieties, instead they may be hybrids among the four botanical varieties [25].

### 2.2. Phenotypic Statistics

A total of 165 key germplasms of the 195 analyzed peanut accessions were planted at three different locations (Dongying, Juxian, and Laixi) in May in China during 2013, 2014, and 2016. Each accession was represented by 34–40 plants that were grown in a two-row plot (5.00 m long and 0.80 m wide). Seven different yield-related traits were evaluated for each accession: Hundred-seed weight (SW), hundred-pod weight (PW), yield per plant (YP), mature pod number per plant (MPP), pod number per plant (PNP), pod branch number per plant (PBP), and total branch number per plant (TBP). Each yield-related trait for each harvested accession was repeatedly measured three times for each study location and year, and these three measurements were then averaged out, so in total, nine mean values (3 locations × 3 years) for each trait of each accession were acquired. To minimize environmental effects, one BLUP (best linear unbiased prediction) value were estimated for each trait of each accession from the nine mean values obtained above, and it was these BLUP values that would be used later on in the GWAS analyses (cf. [26]). The correlation coefficients of each pair of the analyzed traits were calculated with the R function “cor” (https://cran.r-project.org/bin/windows/base/), and the broad-sense heritability (*H*^2^) for each trait was estimated using the R package “lem4” [27].

### 2.3. Genotyping by Sequencing of Peanut Cultivars

Fresh leaves per accession were collected at Shandong Peanut Research Institute, Shandong Academy of Agriculture Science, and it was from these leaves that DNA was extracted using the DNeasy Plant Mini Kit (QIAGEN, Beijing, China). The extracted DNA was firstly electrophoresized and visualized in agarose gel containing Super GelRed (US Everbright Inc., Suzhou, China), and then had their quality and concentration measured by the Nanodrop™ 2000 spectrophotometer (Thermo Scientific, Shanghai, China) and the Qubit^®^2.0 fluorometer (Thermo Scientific, Shanghai, China). Each DNA sample has a concentration of no less than 50 ng/μl and a total DNA of more than 2 μg.

The genotyping by sequencing (GBS) libraries were constructed using a double digest GBS approach that employed a rare–common pair of restriction enzymes [28,29] (*EcoR* I and *Nia* III, New England Biolabs, Ipswich, MA, USA) to perform digestion reactions on the above-prepared DNA samples [30]. The digested products first had both of the DNA ends ligated with A1 or A2 adapters separately and were then pooled together to produce the libraries for all the 195 studied peanut accessions. From the pooled libraries, 350 bp DNA fragments were separated effectively on a 1% agarose gel, column-cleaned using PCR purification kit (New England Biolabs), and amplified for 12 cycles using Phusion DNA polymerase (New England Biolabs). The constructed GBS libraries have their concentration adjusted to 10 nmol/L and sequenced on an Illumina HiSeq Xten platform (Illumina, Guangzhou, China).

The raw reads generated from Illumina sequencing were filtered to get high-quality reads by removing adapter contamination, reads with ≥10% unidentified nucleotides, and reads with >50% low Phred scores (≤ 10). The acquired high-quality reads were then mapped onto a peanut reference genome (https://www.peanutbase.org/data/public/Arachis_hypogaea/Tifrunner.gnm1.KYV3/) using BWA v0.6.2 (-t 4 –M –k 32 –r 1 –c 1) [31].

SNP calling was performed for all samples using the GATK’s Unified Genotyper (https://software.broadinstitute.org/gatk) [32]. The identified SNPs were filtered to reduce the false positive errors using GATK Variant Filtration. To improve SNP data quality, the SNP candidates were further filtered based on the following criteria: (i) Quality score >2.0; (ii) coverage depth >3 fold; (iii) missing ratio within each population <20%; (iv) a global minor allele frequency (MAF) >0.05. All high-quality SNPs were annotated using ANNOVAR [33].

### 2.4. Population Genetic Analysis

Based on the SNPs that were identified above, a phylogenetic tree was constructed by a neighbor-joining (NJ) method as implemented in PHYLIP v3.69 [34], with the bootstrap values calculated with 10,000 replicates. The population structure of the analyzed samples was first preliminarily inferred with the principal component analysis (PCA) incorporated in the software package GCTA (genome-wide complex trait analysis) [35] and was then further analyzed using Admixture v1.3.0 [36]. For the Admixture analysis, 10 potential numbers (1 to 10) of ancestral populations (*K*) were tested using a cross-validation procedure, and the one with the lowest cross-validation error was chosen as the best *K* value (http://software.genetics.ucla.edu/admixture/admixture-manual.pdf) [36]. The matrix of pairwise kinship coefficients among the studied accessions was calculated using the software SPAGeDi v1.5 [37].

### 2.5. Genome-Wide Association Study Analysis

Genome-wide association study (GWAS) analyses of the seven aforementioned yield-related traits based on the acquired high-quality SNPs were conducted using TASSEL v5.0 [38,39]. Four different models were tried for each trait: A general linear model that focuses only on the SNP effect (here referred to as the GLM model), a second general linear model that also takes population stratification (represented by the Q-matrix of ancestry coefficients) into consideration (the Q model), a mixed linear model that considers degree of genetic covariance among the studied individuals (estimated as the K-matrix of relative kinship coefficients) instead of population stratification (the K model), and another mixed linear model that includes both the population and family structure effects (here referred to as the MLM model). The Q-matrix was estimated using Admixture v1.3.0 with the most likely numbers (i.e., *K* = 4) of ancestral populations, while the K-matrix was acquired from SPAGeDi v1.5, and both matrices are based on all the high-quality SNPs identified in the present study. For each trait, the best model out of the tested four based on Q-Q plot was accepted as the final model. We used Bonferroni corrected *p*-value to take the multiple testing problems into consideration, and a *p*-value of 0.05/(the total number of SNP markers) (i.e., 0.05/13435 = 3.72 × 10^−6^) or less was required to establish the significance. A candidate genome region that may be responsible for the studied yield-related traits was defined as the most strongly associated SNP (thereafter referred to as peak SNP) plus a 200kb-long genomic region that centered on the peak SNP [10,40].

### 2.6. Candidate Gene Identification

The candidate genome regions identified above were compared with the QTLs from previous relevant QTL/GWAS mapping studies [6,7,8,9,12,13,14], and the candidate genomic regions that co-localized with QTLs from those earlier studies received close examination. The genes within the candidate genomic regions were defined as candidate associated genes (CAGs). Both gene ontology (GO) enrichment analysis and KEGG (Kyoto Encyclopedia of Genes and Genomes pathway database) pathway enrichment analysis were carried out on the CAGs using the Omicshare web server (www.omicshare.com/tools), and provided information about which biological processes/KEGG pathways these genes were enriched in. To accommodate the multiple testing problem in the enrichment analyses, FDR adjusted *p*-values were calculated with a value of 0.05 being the significance threshold.

## 3. Results

### 3.1. Characterisation and Distribution of SNPs in The Peanut Genome

To reveal the genetic base of seven yield-related traits, a total of 195 peanut accessions were genotyped using the genotyping-by-sequencing (GBS) approach. Sequencing of the GBS libraries produced approximately 1695 million clean reads, which were of high quality (93.1% reads with a Phred score >30) and had a GC content of 37.6–40.2%. About 98.88% of those clean reads were successfully mapped to the peanut reference genome. These clean reads were deposited in the sequence read archive database under SRA accession: PRJNA525244.

**Figure 1:**
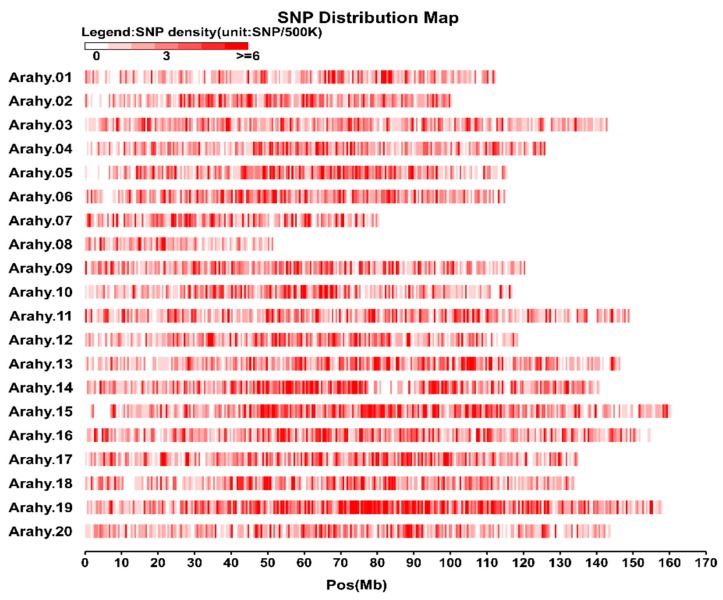
Single nucleotide polymorphisms (SNP) distribution in the 20 chromosomes of the cultivated peanut. The horizontal axis shows chromosome length (Mb), the shades of red represent SNP density (the number of SNPs per window). The vertical axis shows the 20 chromosomes.

Following a stringent SNP calling protocol, a total of 13,435 SNPs were identified (Table S3). The peanut genome had an average SNP density of 5.93 SNPs/Mb with the densities at chromosomes Arahy.08 (3.70 SNPs/Mb) and Arahy.19 (7.45 SNPs/Mb) being, respectively, the lowest and the highest (Figure 1; Table S4). Most of the identified SNPs were found at intergenic regions (89.9%), while the exonic, intronic, up-, and down-stream regions only accounted for, respectively, 3.1%, 2.6%, and 3.6% of the total SNPs (Table S5). Of those SNPs within exonic regions, 35.06% were nonsynonymous while 62.07% were synonymous. The genome-wide transition/transversion (*Ts/Tv*) ratio for the analyzed peanut genome data was 1.94 (Figure S2).

### 3.2. Genetic Diversity, Population Structure, and Genetic Diversity

Overall, the levels of genome-wide nucleotide diversity (π) of the irregular accession group (π = 0.00042) being higher than those of the four studied peanut botanical varieties (π = 0.00006–0.00025). To be specific for the botanical varieties, the highest level of nucleotide diversity (π = 0.00025) was found in *A. hypogaea* var. *hirsuta* while the lowest (π = 0.00006) in var. *hypogaea*, with those of var. *fastigiata* (π = 0.00011) and var. *vulgaris* (π = 0.00018) being in the middle.

**Figure 2:**
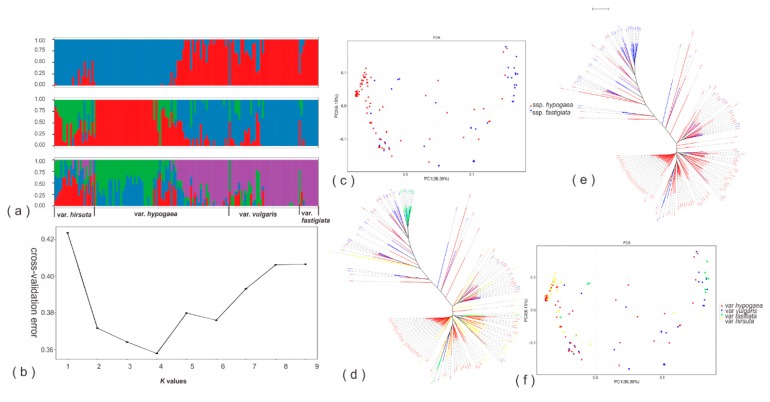
Genetic diversity and population structure of the studied peanut accessions. (**a**) Population structure, each accession is represented by a single vertical line and colors represent ancestries. (**b**) Estimated Ln (probability of the data) calculated for each K ranging from 1 to 9. (**c**,**f**) Scatter plots of the first two principal components (PCA analyses), each dot represents one accession. (**d**,**e**) Phylogenetic trees constructed using the neighbor-joining method.

A neighbor-joining (NJ) tree inferred from the acquired SNPs showed that the analyzed peanut accessions could be approximately classified into two major groups, with the first group being dominated by samples from *A. hypogaea* ssp. *hypogaea* (var. *hypogaea* and var. *hirsuta*), while the second group mostly comprised accessions from *A. hypogaea* ssp. *fastigiata* (var. *vulgaris* and var. *fastigiata*), but also with a considerable proportion of members coming from ssp. *hypogaea* (mostly var. *hypogaea*) (Figure 2e,d).

Results from the principal component analysis (PCA) were in accordance with those of the phylogenetic analysis (Figure 2c,f). The population structure of the studied peanut samples was further investigated by the Admixture software. Out of the 10 tested potential numbers (*K*: 1–10) of ancestral populations, *K* = 4 represented the most sensible choice according to cross-validation error value: It has the lowest value (Figure 2b), however its difference with *K* = 3 was small (Figure 2b). For *K* = 4, the first three ancestral populations (green, blue, and red, Figure 2a) were dominated by individuals from *A. hypogaea* var. *hirsuta* and var. *hypogaea*, while the fourth ancestral population (purple) prevailed the genetic makeups of var. *vulgaris*, var. *fastigiata* and ca. 40% of var. *hypogaea*. For *K* = 3, again var. *hirsuta* and a big part of var. *hypogaea* dominated the first two ancestral populations (green and red) while the rest of the peanut accessions constituted the majority of the third population (blue). Overall, the results of both *K* = 3 and 4 agreed well with that of *K* = 2; for the latter, the first population was mostly composed of individuals from var. *hirsuta* and a big part of var. *hypogaea*, whereas the second ancestral population had var. *vulgaris*, var. *fastigiata* and ca. 40% of the var. *hypogaea* individuals as its main members.

### 3.3. Phenotypic Correlation and Heritability for Different Traits

**Figure 3:**
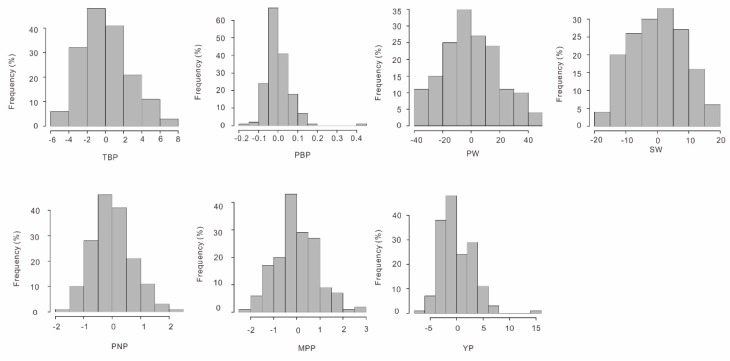
The frequency distribution of the studied peanut yield-related traits.

One hundred and sixty-five out of the 195 studied peanut accessions have been phenotyped for three years at three different locations, and based on these phenotype data, the correlation and the heritability of seven agronomic traits (hundred-seed weight (SW), hundred-pod weight (PW), yield per plant (YP), mature pod number per plant (MPP), pod number per plant (PNP), pod branch number per plant (PBP), and total branch number per plant (TBP)) were estimated. All seven traits were found to follow the normal distribution without any significant skewness and kurtosis (Figure 3). The correlation between the seven traits were all positive, with the correlation coefficient (*r*) estimates being, or less than, 0.45, except those for the trait pairs YP–PBP/MPP/SW/PW (0.60–0.66), as well as SW–PW (0.91) and MPP–PBP (0.93) (Figure S3). The broad-sense heritability (*H^2^*) for SW (*H*^2^ = 0.72) and PW (0.63) were the highest among the seven agronomic traits, while those for PBP (0.04), PNP (0.14), and MPP (0.19) were the lowest, and those for TBP (0.54) and YP (0.39) were in the middle (Table 1).

### 3.4. Genome-Wide Association Studies in Peanut

**Figure 4:**
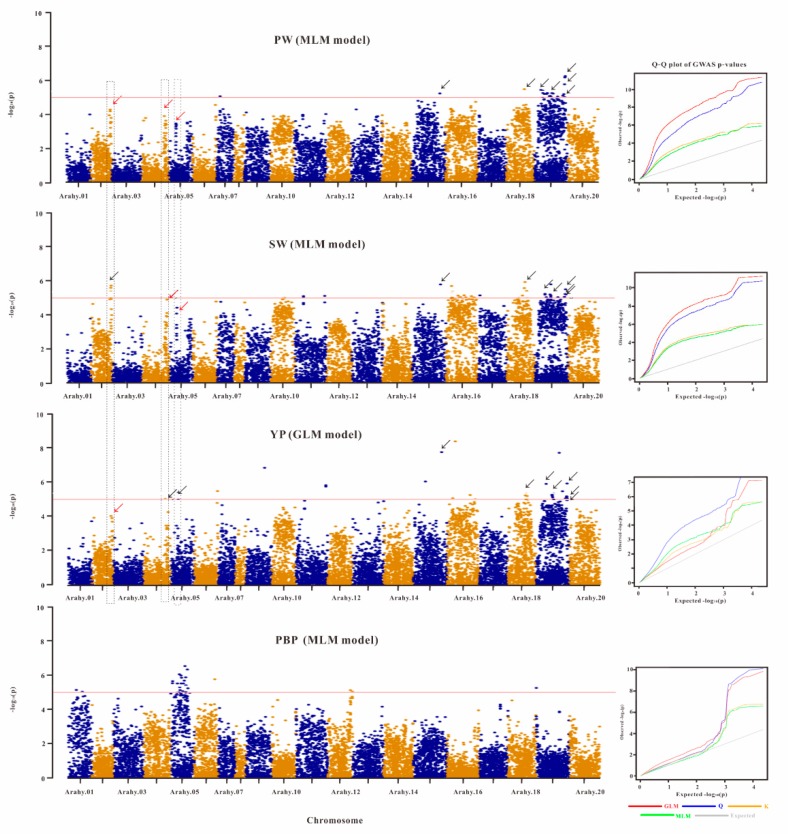
Manhattan plots showing the associations of all SNPs with four yield-related traits. The four yield-related traits are, respectively, hundred-pod weight (PW), hundred-seed weight (SW), yield per plant (YP), and pod branch number per plant (PBP). For each trait, the Q–Q plots from four different statistical models are also shown in the right. The shown Manhattan plots are from the best statistical models (according to Q–Q plots). On chromosomes Arahy.15, Arahy.18, and Arahy.19, there are, respectively, one, one, and five SNPs (see the black arrows in these regions) that are associated with PW, SW, and YP. On chromosomes Arahy.02, Arahy.04 and Arahy.05, the dashed blocks enclose, respectively, one SNP cluster where the top SNP is significantly (black arrow) or not significantly (red arrow) associated with traits SW, PW, and YP. The significance level is log10 (0.05/13435) = 5.3 (the red horizontal line).

Genome-wide association analyses of the phenotypically characterized peanut accessions were conducted to see if any of the acquired SNPs had variants that were associated with the considered yield-related traits. Significantly associated SNPs were found for four of the studied agronomic traits (SW, PW, YP, and PBP), with the MLM and GLM models being the best statistical models, respectively, for traits SW, PW, PBP, and for trait YP based on the Q–Q plots (Figure 4). It should be noted that there is considerable residual inflation in the Q–Q plots for the best models of traits PW and SW, therefore the associated SNPs identified for these two traits should be interpreted with caution.

For SW that was estimated to show the highest heritability (*H*^2^ = 0.72) among the considered traits, a total of 38 peak SNPs were found to be significantly associated with it (*p*-value < 3.72 × 10^−6^) (Table S6). Three of these SW-associated peak SNPs were located on chromosome Arahy.02, one on Arahy.10, three on Arahy.11, one on Arahy.15, 12 on Arahy.16, one on Arahy.17, five on Arahy.18, and 12 on Arahy.19 (Figure 4). For PW that showed the second highest heritability (*H*^2^ = 0.63), 23 significantly associated peak SNPs (*p*-value < 3.72 × 10^−6^) have been identified from chromosomes Arahy.07 (1 SNP), Arahy.15 (1 SNP), Arahy.18 (1 SNP), and Arahy.19 (20 SNPs), and it is worth to be noted that the *p*-value for one peak SNP on Arahy.19 was < 10^−6^ (Figure 4; Table S6). For YP that had a relatively lower heritability (*H*^2^ = 0.39) compared to SW and PW, 29 associated peak SNPs (*p*-value < 3.72 × 10^−6^) had been identified from chromosomes Arahy.04 (1 SNP), Arahy.05 (2 SNP), Arahy.06 (1 SNP), Arahy.09 (1 SNP), Arahy.11 (2 SNPs), Arahy.15 (2 SNPs), Arahy.16 (4 SNPs), Arahy.18 (3 SNPs), and Arahy.19 (13 SNPs). On each of the chromosomes Arahy.09, Arahy.15, Arahy.16, and Arahy.19, one peak SNP was found to have a *p*-value smaller than 10^−6^. In addition, 26 significant peak SNPs that were significantly associated with PBP (*H^2^* = 0.04) were found on chromosome Arahy.01 (2 SNPs), Arahy.05 (20 SNPs), Arahy.06 (1 SNP), Arahy.12 (2 SNPs), and Arahy.19 (1 SNP). Among these identified SNP peaks, rs9144 on chromosome Arahy15, rs11390 on chromosome Arahy18, as well as rs11866, rs12090, rs12695, rs12720, and rs12730 on chromosome Arahy.19 were all associated with traits SW, PW, and YP simultaneously.

To sum it up, there were 93 non-overlapping peak SNPs that had been identified to be associated with the studied yield-related traits, and annotation of the 200kb genome regions that centered on these peak SNPs found a total of 311 unique preliminary candidate associated genes (CAGs) (Table S7).

### 3.5. Gene Ontology (GO) and Kyoto Encyclopedia of Genes and Genomes (KEGG) Pathway Enrichment Analyses

Gene annotation of the 200kb genomic regions that centered on the peak SNPs, which had been identified to be associated with the studied yield-related traits, found 107 candidate associated genes (CAGs) for trait PBP, 70 CAGs for PW, 132 CAGs for SW, and 88 CAGs for YP (Table S7). GO enrichment analyses of these candidate genes revealed that there were in total 24 GO processes that were significantly enriched among the PBP-associated CAGs, with hydrogen ion transmembrane transport (GO: 1902600) and glucose metabolic process (GO: 0006006) being the two most significant ones (Table S7). There were 33 GO processes significantly enriched among PW-associated CAGs, with chlorophyll biosynthetic process being the most significant one. For SW, there were only three significantly enriched GO processes: Non-recombinational repair (GO: 0000726), DNA repair (GO: 0006281), and cellular response to DNA damage stimulus (GO: 0006974). No GO process is significant for YP. 

Regarding the KEGG pathway enrichment analyses, the two significant pathways enriched in the CAGs for PBP were autophagy (ko04136, Cellular processes) and oxidative phosphorylation (ko00190, Metabolism). There was only one significantly enriched pathway (ko03020, RNA polymerase) for PW (genetic information processing). For SW, four pathways were significantly enriched among the associated CAGs: Non-homologous end-joining (ko03450) and proteasome (ko03050) (both were Genetic information processing), as well as tyrosine metabolism (ko00350) and nitrogen metabolism (ko00910) (both belonging to Metabolism). No pathway was significant for YP (Table S7).

### 3.6. Literature Survey of Previous Identified QTLs

The previous QTL mapping/GWAS studies (using different sets of peanut accessions comparing to the present study) that identified significant QTL regions for traits PBP, SW, PW, and YP have been summarized (Table 2 and Table S8), and the QTL physical intervals of these identified QTLs for each trait were then determined based on the available left/right marker primer sequences (Table S8). From these earlier QTL/GWAS studies, 2, 46, 29, and 35 matched genomic regions were, respectively, found to be associated with traits PBP, PW, SW, and YP (Table S8). 

**Figure 5:**
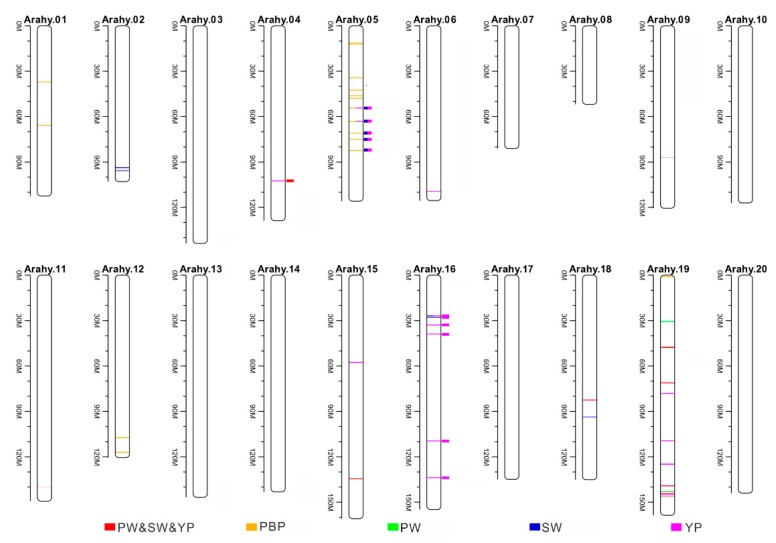
Quantitative trait loci (QTLs) identified to be associated with yield-related traits by both the current GWAS and earlier QTL mapping studies. The orange, green, blue, and magenta colors, respectively, for the yield-related traits PBP, PW, SW, and YP. The colorful lines represent the QTLs identified by the present study, the solid squares point to the QTLs that are co-localized with earlier identified QTLs. Most of these co-localized QTLs are located on chromosomes Arahy.05 and Arahy.16.

Theses earlier reported QTLs for traits SW, PW, YP, and PBP were then compared with the genomic regions that had been identified to be associated with the same traits in the present GWAS study. None of the earlier reported QTLs for PBP were co-located with those identified in the present study. However, a total of 12 co-localized genomic regions were found for traits SW (six, corresponding peak SNP codes: rs2675, rs2560, rs2639, rs2500, rs2123, rs2725), PW (one, peak SNP code: rs2123), and YP (12: rs2675, rs2560, rs2639, rs9411, rs2500, rs9380, rs2123, rs2725, rs9432, rs9906, rs9379, rs10035), among which one was located on chromosome Arahy.04 (rs2123), five on Arahy.05 (rs2675, rs2560, rs2639, rs2500, rs2725), and six on Arahy.16 (rs9411, rs9380, rs9432, rs9906, rs10035, rs9379) (Figure 5 and Table 2). It is worth to be noticed that all these co-localized earlier reported QTLs were from QTL mapping (not GWAS) studies.

Gene annotation of the 12 peak SNPs that were co-localized with earlier identified QTLs found a total of 36 CAGs. KEGG pathway enrichment analyses showed that these 36 CAGs were enriched in 19 different KEGG pathways (Table S9), however none were significant (corrected *p* value < 0.05). Ten of the enriched pathways were involved in metabolic pathways (e.g., nitrogen metabolism, fatty acid biosynthesis, and fatty acid degradation) (Table S9). GO enrichment analysis of these 36 CAGs found no significantly enriched GO process, but one of the top 10 processes is the nucleotide–sugar metabolic process (GO: 0009225). However, one should interpret this result with caution, because the number of genes that were analyzed is very limited (only 36), and only six of these genes were, respectively, recognized/considered by the GO and KEGG analyses.

## 4. Discussion

The cultivated peanut is an important oilseed crop, which is widely cultivated across tropical, subtropical, and warm temperate area [41,42]. It originated in South America, from where it spread around the world and at the same time evolved phenotypically and genotypically, which allows it to adapt to various agro-ecological environments [42,43]. In the present study, we have analyzed 195 peanut accessions, which represent the majority of the Chinese peanut landraces and encompass rich genetic variations [22]. China is the largest producer and exporter of peanut in the world [2,42]. Based on the acquired genotype and phenotype data, we carried out genome-wide association analyses with the aim to discover the genetic basis for several yield-related traits [17,44].

### 4.1. Genome-Wide SNP Discovery

The genotyping in this study was performed with the genotyping-by-sequencing approach that uses genome sequencing (with reduced genome representation, but on multiplexed samples) to complete the genome-wide molecular marker (SNP) discovery and genotyping at the same time [29]. This high-throughput genotyping approach is considered to be efficient, reliable, and cheap [15,45,46]. The (tetraploid) cultivated peanut comprises AA and BB sub-genomes, which are closely related to each other, and transcriptome assembly of cultivated peanut has been shown to be challenging due to the difficulty in separating the A and B sub-genome gene sequences [41]. To avoid mixing the homologous regions between these two sub-genomes, a very stringent SNP-calling procedure has been adopted, especially when mapping the high-quality reads onto a reference genome using BWA, only one mismatch (-r 1) for each read is allowed; this strategy may filter out a large number of SNPs, but make the identified SNPs and their genome locations highly confident. In total, 13,435 SNPs are identified from the 195 peanut accessions and consistent with Zhang et al. [10], these SNPs are not evenly distributed on the 20 chromosomes of the domesticated peanut, with the B sub-genome (Arahy.11–Arahy.20) containing more SNPs (8134) than the A sub-genome (Arahy.01–Arahy.10) (5359) (Figure 1 and Table S4). The genome-wide average SNP density is 5.93 SNPs/Mb, which is comparable with a relevant earlier result (seven SNPs/Mb in [10]) and acceptable for GWAS analyses in peanut (cf. [10]). In addition, our results show that there is a higher frequency of transitions over transversions within peanut genomes (ratio = 1.94), which is consistent with earlier observations from other species, e.g., rice [47], maize [48], potato [49], pepper [50], and soya bean [18]. This “transition bias” is due to the conformational similarity between purines (A, G) (or between pyrimidines (T, C)), mis-pairing caused by transitional mutations; (C
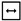
T, A
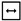
G) is therefore conformationally more favorable than that generated by transversions (A
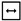
C, C
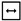
G, A
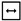
T, G
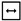
T) [51]. Moreover, transitions give rise to less amino acid replacements and tend to conserve the chemical property of an amino acid if it does bring on amino acid replacements.

### 4.2. Population Structure

The domesticated peanut has been grouped into two subspecies (ssp. *hypogaea* and ssp. *fastigiata*), which can be further sorted out into six botanical varieties [44]. The 195 studied peanut accessions in this study are mostly samples from four botanical varieties (ssp. *hypogaea*: var. *hirsuta* and var. *hypogaea*; ssp. *fastigiata*: var. *vulgaris* and var. *fastigiata*) and almost equally represent the two subspecies (Figure 2). Our PCA, phylogenetic, and Admixure analyses that are based on genomic data also reveal two major groups within the domesticated peanut, and overall, these two groups agree with the taxonomic grouping, except some (ca. 40%) var. *hypogaea* individuals (Figure 2). One major group corresponds to ssp. *hypogaea* and comprises mostly individuals from var. *hirsuta* and var. *hypogaea* (ca. 60% of the individuals from this species), while the other major group more or less matches up with ssp. *fastigiata* and is composed of individuals from var. *vulgaris* and var. *fastigiata*, as well as ca. 40% of the var. *hypogaea* individuals. Perhaps the unexpected behaviour of the ca. 40% of the var. *hypogaea* individuals that are genetically more closely related to ssp. *fastigiata* instead of ssp. *hypogaea* (Figure 2) is not surprising, because similar ambiguous genetic boundary between the two domesticated peanut subspecies have been reported in other studies [10,15] as well, even though different sets of peanut accessions that represent the two subspecies differently were used in these studies. These unexpected behaviors may suggest genetic introgression between ssp. *fastigiata* and ssp. *hypogaea* during artificial selection [52,53]. In addition, all the analyzed 195 peanut accessions are recently diverged, and it has also been shown that more than 40% of Chinese peanut cultivars originated from a limited number of elite germplasms (e.g., ‘Fuhuasheng’, ‘Xuzhou68-4’, ’Shitouqi’, or ‘Yueyou551’) [2,54,55], therefore it may be hard to avoid that the shared ancestral polymorphism complicates the genetic delimitation among subspecies/cultivars.

### 4.3. Yield-Related Candidate Genes

Our GWAS analyses have identified a total of 93 non-overlapping SNP peaks that are associated with four yield-related traits, and seven peak SNPs were identified to be associated simultaneously with three traits (YP, PW, SW), which is not surprising considering that traits YP, SW, and PW are highly correlated with each other (r > 0.6, Figure S3). Genes responsible for the yield-related traits may include or be linked to the identified SNP peaks. In order to look for candidate genes, the 200 kb genomic regions that centered on, and are also very likely to be linked to [10], these SNP peaks were annotated; a total of 311 unique candidate associated genes (CAGs) were identified. However, it should be noted that because there is considerable residual inflation in the Q–Q plots for the best models of traits PW and SW (Figure 4), which may be due to unknown factors that were not considered in the models, the CAGs identified for these two traits should be interpreted with caution. Among the 311 unique identified CAGs, 36 are from genomic regions surrounding the 12 SNP peaks (co-localized genomic regions, on chromosomes Arahy.04, Arahy.05, and Arahy.16) that have been identified to be associated with yield-related traits in peanut by not only the present study but also earlier QTL mapping studies (Table 2 and Table S9). It should be mentioned that five (arahy.7B5I5W, arahy.KBV6L0, arahy.Y4GE00, arahy.A3WBL0, and arahy.H97NC3) of these 36 CAGs surround two SNP peaks (rs9379 and rs9380) that are associated with trait SW, and none of the 36 CAGs are for trait PW. 

A close examination of these 36 CAGs around the 12 relatively confident QTLs for yield found that the *arahy*.*RI9HIF* gene (predicted to produce high affinity nitrate transporter 2.4 in *A. hypogaea*) (within the 200 kb genomic region centering on the SNP peak, rs9411, on chromosome Arahy.16) may deserve more attention. This is because one rice homolog (*OsNRT2.3*) of this gene produces a protein (*OsNRT2.3b*) that has been shown to improve yield in rice when over expressed [56,57]. Whether variation at *arahy.RI9HIF* or at some other CAGs especially in the co-localized genomic regions significantly influences peanut yield needs further investigation.

## 5. Conclusions

By carrying out GWAS analyses, we have identified 93 non-overlapping SNP peaks that are significantly associated with four yield-related traits in peanut, and gene annotation of the genomic regions surrounding these SNP peaks identified 311 unique candidate genes. A comparison with earlier related QTL mapping/GWAS studies show that 12 of the 93 yield-related-trait-associated SNP peaks are co-localized with earlier identified QTLs, and 36 candidate genes have been identified from these 12 co-localized genomic regions. 

## Figures and Tables

**Table 1:** Phenotypic statistics of peanut yield-related traits.

Traits	Abbr.	Maximum	Minimum	Median	Average	Variance	SD	CV (%)	H^2^
Total Branching Number	TBN	31.27	9.26	18.34	18.80	19.81	6.28	33.00	0.54
Pod Branching Number	PBP	16.16	4.84	7.93	8.07	1.57	1.25	16.00	0.04
Pods Number Per Plant	PNP	38.97	15.58	24.16	24.46	17.19	4.15	17.00	0.14
Mature Pods Number Per Plant	MPP	31.89	10.34	19.02	19.38	14.60	3.82	20.00	0.19
100-Pod Weight	PW	235.16	100.73	159.24	160.55	859.34	29.31	18.00	0.63
100-Seed Weigh	SW	84.54	35.93	59.55	59.20	125.03	11.18	7.00	0.72
Yield Per Plant	YP	60.86	12.12	25.34	26.95	47.54	6.90	26.00	0.39

NOTE: SD, standard deviation. CV, coefficient of variance. H^2^, broad-sense heritability.

**Table 2:** A summary of the genomic regions that are co-localized with earlier identified QTLs.

The Present Study				Earlier Studies		
Peak SNP	Position (± 100 kb)	Associated Traits	Annotated Gene List	Co-localized QTLs^a^	Studied Traits	Ref.
**rs2123**	Arahy.04: 102215951	YP	arahy.Q2K3EA; arahy.JMQ6FC	Arahy.04: 94872059-124685617	PW;SW;YP	[1]
**rs2500**	Arahy.05: 54266788	PBP; YP	arahy.T4811Q; arahy.71GWZM	Arahy.05: 47955696-84171615;	YP; SW	[23]
**rs2560**	Arahy.05: 63009987	PBP; YP	arahy.A6MV6T	Arahy.05: 47955696-84171615; 6445048-6913313	YP; SW	[1]
**rs2639**	Arahy.05: 70800888	PBP	arahy.1NMA8N; arahy.J02DSU	Arahy.05: 47955696-84171615;	YP; SW	[23]
**rs2675**	Arahy.05: 75058945	PBP	arahy.7FT54I; arahy.D7MP66; arahy.V7ESBJ	Arahy.05: 47955696-84171615;	YP; SW	[1]
**rs2725**	Arahy.05: 82267261	PBP	arahy.LH3A9J; arahy.NM6FQB; arahy.AVNN4W	Arahy.05: 47955696-84171615;	YP; SW	[23]
**rs9379**	Arahy.16: 26887159	SW, YP	arahy.7B5I5W	Arahy.16: 16836027-41668600	YP	[23]
**rs9380**	Arahy.16: 26954799	SW	arahy.KBV6L0; arahy.Y4GE00; arahy.A3WBL0; arahy.H97NC3	Arahy.16: 16836027-41668600	YP	[23]
**rs9411**	Arahy.16: 32888295	YP	arahy.RI9HIF; arahy.HCD6SP; arahy.9BIX4A; arahy.N88R54	Arahy.16: 16836027-41668600	YP	[23]
**rs9432**	Arahy.16: 38878752	YP	arahy.SDZN59; arahy.KW19LS; arahy.FC0UQU; arahy.PY3JPZ; arahy.05RLXM; arahy.K0A92D	Arahy.16: 16836027-41668600	YP	[23]
**rs9906**	Arahy.16: 109497037	YP	arahy.60591L	Arahy.16: 16836027-141668600	YP	[23]
**rs10035**	Arahy.16: 133839995	YP	arahy.84A022; arahy.V06H0G; arahy.C96CVE; arahy.7GY2ZZ; arahy.A4HJXM; arahy.QZI7QE; arahy.AA3MKH	Arahy.16:16836027-141668601	YP	[23]

^a^ The physical intervals of the co-localized QTLs have been determined based on the available left/right marker primer sequences (Table S8).

## Supplementary Materials

The following are available online at https://www.mdpi.com/2073-4425/10/10/803/s1, Figure S1: Geographic distribution of the analyzed peanut accessions. Each accession is displayed as a dot. Different colors stand for the different botanical varieties. (NOTE: The map used here represents only part of China), Figure S2: A summary of the transitions and transversions that occurred within the acquired SNPs (*T*s/*T*v = 1.94), Figure S3: Correlation coefficients between the studied phenotypic traits, Table S1: Basic information on the 195 studied peanut accessions, Table S2: A summary of the predicted genome-wide restriction reaction result using the enzyme pair *EcoR* I and *Nia* III, Table S3: Match statistics for the BWA mapping of the acquired high-quality reads to a peanut reference genome and the number of acquired SNPs after different filtering steps, Table S4: The distribution and frequency of the identified SNPs on the 20 peanut chromosomes, Table S5: A summary of the distributions of the acquired SNPs in different genic and intergenic regions, Table S6: The peak SNPs that are associated with the yield-related traits PBP, PW, SW, and YP under the respective best model for each traits, Table S7: Information on the annotated genes from the 200kb genome regions centered on the peak SNPs that are, respectively, associated with traits PBP, PW, SW, and YP, Table S8: Information on 199 earlier identified related QTLs and their physical locations. These QTLs were found to be associated with the yield-related traits PW, SW, YP, or PBP, Table S9: Information on 36 candidate genes that are identified, by the present GWAS study, to be associated with yield traits SW, YP, and PW and are at the same time co-localized with earlier identified QTLs.
