# Supplementary material for: GWAS Discovery of Candidate Genes for Yield-Related Traits in Peanut and Support from Earlier QTL Mapping Studies
Source: Genes (Basel). 2019 Oct 12;10(10):803. doi: 10.3390/genes10100803 (PMC6826990; doi:10.3390/genes10100803)
Supplement: Supplementary file 1 [file genes-10-00803-s001.zip › Table S2-S5.docx]

**Table S2.** A summary of the predicted *g*enome-wide restriction reaction result using the enzyme pair *EcoR* I and *Nia* III.

| **All Tags length (bp)** | **All Tags coverage** | **All RF ave-length (bp)** | **Effective Tags number** | **Effective Tags length (bp)** | **Effective Tags coverage** |
| --- | --- | --- | --- | --- | --- |
| 293,401,200 | 0.1231 | 267 | 412,662 | 61,899,300 | 0.0246 |

**Table S3.** Match statistics for the BWA mapping of the acquired high-quality reads to a peanut reference genome and the number of acquired SNPs after different filtering steps.

| **Feature** | **Value** |
| --- | --- |
| **Match statistics** |  |
| Mapping rate (%) | 98.88 |
| Average depth | 5.7× |
| **Total number of SNPs** | 361,531 |
| SNPs with quality score >2.0; The average depth of reads $>$3 and <100; MAF$\geq$0.05; 80% coverage | 13,435 |

NOTE：MAF: minor allele frequency.

**Table S4.** The distribution and frequency of the identified SNPs on the 20 peanut chromosomes.

| **Chr.** | **Length (bp)** | **No. of SNPs** | **Kb/SNP** | **SNPs/Mb** |
| --- | --- | --- | --- | --- |
| Arahy.01 | 112,420,854 | 444 | 253 | 3.95 |
| Arahy.02 | 102,981,163 | 537 | 192 | 5.21 |
| Arahy.03 | 143,813,506 | 661 | 218 | 4.60 |
| Arahy.04 | 128,801,742 | 611 | 211 | 4.74 |
| Arahy.05 | 115,930,344 | 629 | 184 | 5.43 |
| Arahy.06 | 115,504,342 | 652 | 177 | 5.64 |
| Arahy.07 | 81,119,488 | 446 | 182 | 5.50 |
| Arahy.08 | 51,897,010 | 192 | 270 | 3.70 |
| Arahy.09 | 120,519,698 | 618 | 195 | 5.13 |
| Arahy.10 | 117,088,237 | 569 | 206 | 4.86 |
| Arahy.11 | 149,299,306 | 743 | 201 | 4.98 |
| Arahy.12 | 120,579,088 | 581 | 208 | 4.82 |
| Arahy.13 | 146,725,006 | 774 | 190 | 5.28 |
| Arahy.14 | 143,237,272 | 864 | 166 | 6.03 |
| Arahy.15 | 160,879,708 | 979 | 164 | 6.09 |
| Arahy.16 | 154,808,347 | 877 | 177 | 5.67 |
| Arahy.17 | 134,922,436 | 800 | 169 | 5.93 |
| Arahy.18 | 135,150,084 | 689 | 196 | 5.10 |
| Arahy.19 | 158,625,764 | 1182 | 134 | 7.45 |
| Arahy.20 | 143,980,330 | 645 | 223 | 4.48 |

**Table S5.** A summary of the distributions of the acquired SNPs in different genic and intergenic regions.

| **Category** | | **Number of SNPs** |
| --- | --- | --- |
| Total |  | 13,435 |
| Exonic | Stop gain | 7 |
|  | Stop loss | 3 |
|  | Synonymous | 122 |
|  | Nonsynonymous | 216 |
| Intronic |  | 487 |
| Splicing |  | 7 |
| UTR3 |  | 54 |
| UTR5 |  | 42 |
| Upstream |  | 193 |
| Downstream |  | 184 |
| Downstream / Upstream (1Kb) |  | 35 |
| Intergenic |  | 12,085 |
