# Supplementary figures and images for: GWAS Discovery of Candidate Genes for Yield-Related Traits in Peanut and Support from Earlier QTL Mapping Studies
Source: Genes (Basel). 2019 Oct 12;10(10):803. doi: 10.3390/genes10100803 (PMC6826990; doi:10.3390/genes10100803)

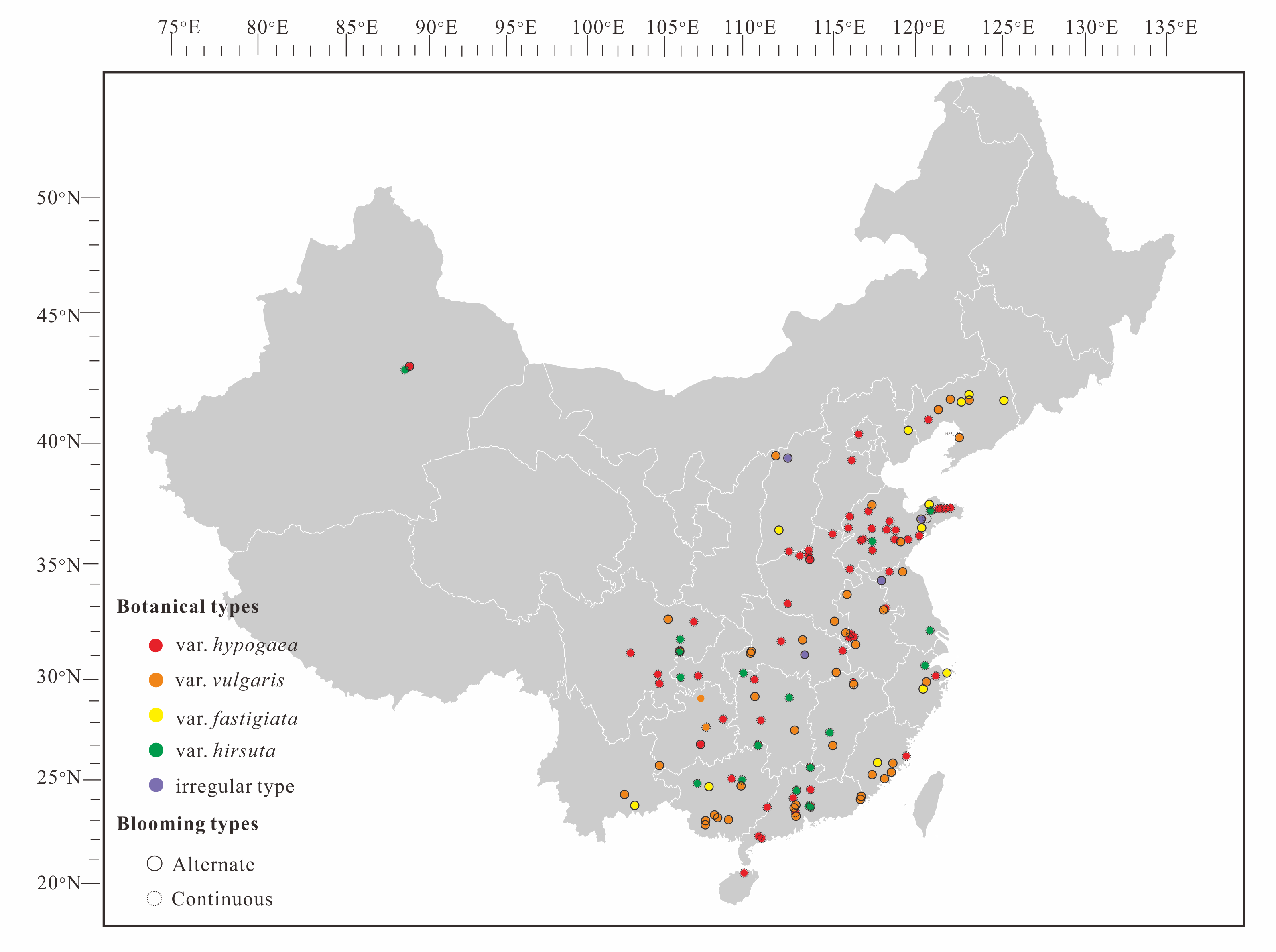

Supplement: Supplementary file 1 [file genes-10-00803-s001.zip › Figure S1.png]

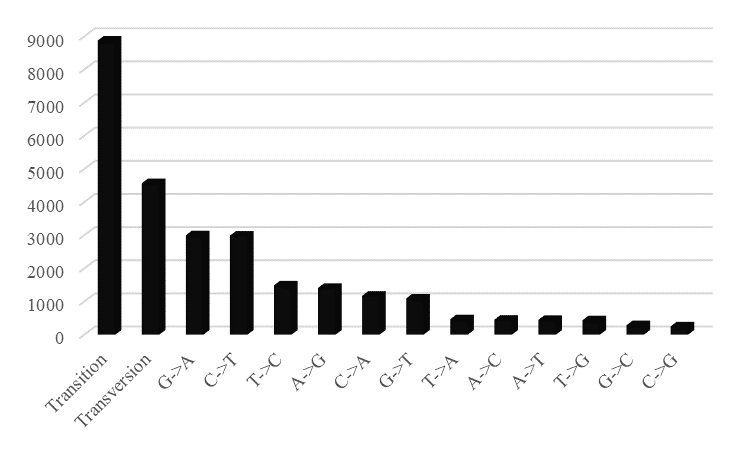

Supplement: Supplementary file 1 [file genes-10-00803-s001.zip › Figure S2.png]

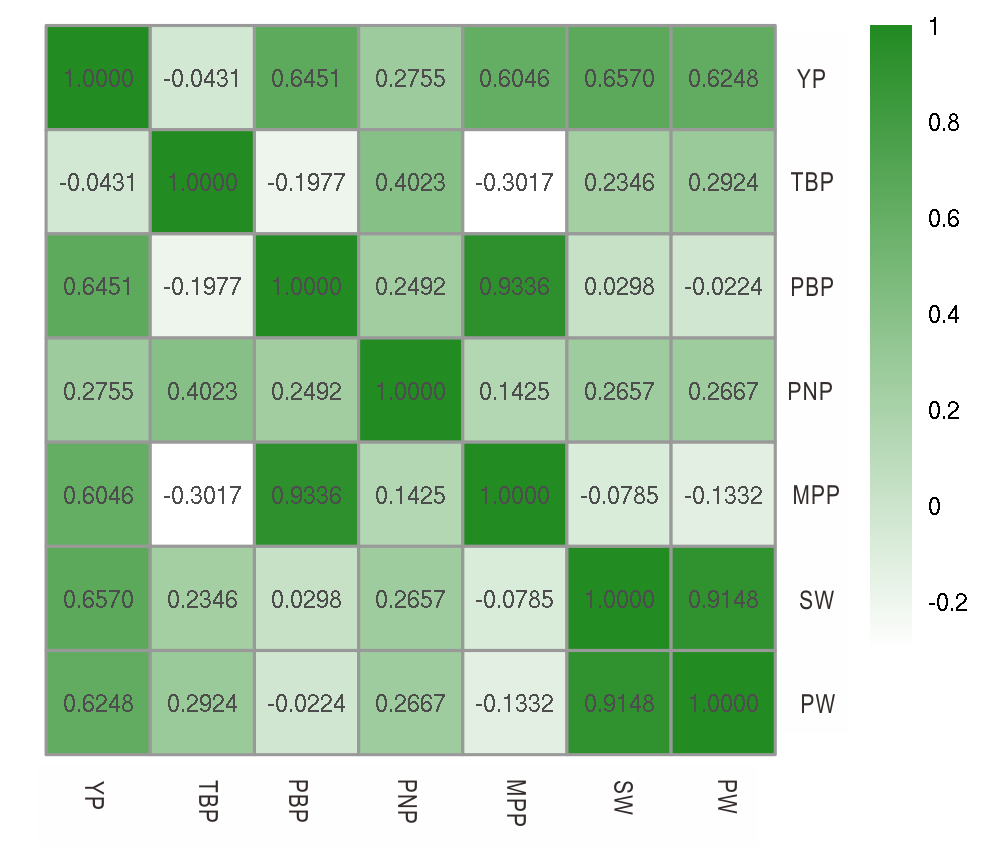

Supplement: Supplementary file 1 [file genes-10-00803-s001.zip › Figure S3.png]
